# Nitrosamine exposure exacerbates high fat diet-mediated type 2 diabetes mellitus, non-alcoholic steatohepatitis, and neurodegeneration with cognitive impairment

**DOI:** 10.1186/1750-1326-4-54

**Published:** 2009-12-24

**Authors:** Suzanne M de la Monte, Ming Tong, Margot Lawton, Lisa Longato

**Affiliations:** 1 Department of Pathology (Neuropathology), Rhode Island Hospital, 593 Eddy Street, Providence, RI 02903 USA; 2 Department of Neurology, Rhode Island Hospital, 593 Eddy Street, Providence, RI 02903 USA; 3 Liver Research Center, Rhode Island Hospital, 55 Claverick Street, Providence, RI 02903 USA; 4 Pathobiology Program, Brown University, Box G, 222 Richmond Street, Providence, RI 02903 USA; 5 Warren Alpert Medical School of Brown University, Box G, 97 Waterman Street, Providence, RI 02912 USA

## Abstract

**Background:**

The current epidemics of type 2 diabetes mellitus (T2DM), non-alcoholic steatohepatitis (NASH), and Alzheimer's disease (AD) all represent insulin-resistance diseases. Previous studies linked insulin resistance diseases to high fat diets or exposure to streptozotocin, a nitrosamine-related compound that causes T2DM, NASH, and AD-type neurodegeneration. We hypothesize that low-level exposure to nitrosamines that are widely present in processed foods, amplifies the deleterious effects of high fat intake in promoting T2DM, NASH, and neurodegeneration.

**Methods:**

Long Evans rat pups were treated with N-nitrosodiethylamine (NDEA) by i.p. Injection, and upon weaning, they were fed with high fat (60%; HFD) or low fat (5%; LFD) chow for 6 weeks. Rats were evaluated for cognitive impairment, insulin resistance, and neurodegeneration using behavioral, biochemical, molecular, and histological methods.

**Results:**

NDEA and HFD ± NDEA caused T2DM, NASH, deficits in spatial learning, and neurodegeneration with hepatic and brain insulin and/or IGF resistance, and reductions in tau and choline acetyltransferase levels in the temporal lobe. In addition, pro-ceramide genes, which promote insulin resistance, were increased in livers and brains of rats exposed to NDEA, HFD, or both. In nearly all assays, the adverse effects of HFD+NDEA were worse than either treatment alone.

**Conclusions:**

Environmental and food contaminant exposures to low, sub-mutagenic levels of nitrosamines, together with chronic HFD feeding, function synergistically to promote major insulin resistance diseases including T2DM, NASH, and AD-type neurodegeneration. Steps to minimize human exposure to nitrosamines and consumption of high-fat content foods are needed to quell these costly and devastating epidemics.

## Background

Alzheimer's Disease (AD), obesity, type 2 diabetes mellitus (T2DM), and non-alcoholic fatty liver disease (NAFLD)/non-alcoholic steatohepatitis (NASH), which includes metabolic syndrome, have sharply increased in prevalence over the past several decades [1-6]. In addition, we have noted striking increases in age-specific AD mortality rates over the same time interval, and correlated these findings with sharply increased consumption of processed foods, use of preservatives, and demand for nitrogen-containing fertilizers [7]. Such rapid shifts in age-adjusted AD morbidity and mortality are more consistent with exposure-related rather than genetic etiologies. A common theme resonating from these lifestyle trends is that we have increased our exposures to nitrosamines (R1N(-R2)-N = O) and related compounds.

Nitrosamines form by chemical reactions between nitrites and secondary amines or proteins, and they exert toxic or mutagenic effects by promoting DNA damage, oxidative stress and reactive oxygen species generation [8]. The end result is increased lipid peroxidation, adduct formation, and pro-inflammatory cytokine activation [9], which also happen to be major mediators of human insulin-resistance diseases, including T2DM, NASH, and AD [10-16]. The notion that chronic injury caused by alkylating agents could lead to malignant transformation and/or tissue degeneration is consistent with the findings that: 1) chronic exposure to tobacco nitrosamines causes both lung cancer and emphysema with chronic obstructive pulmonary disease; and 2) treatment with streptozotocin (STZ), a nitrosamine-related compound, causes hepatocellular carcinoma, pancreatic carcinoma, malignant brain tumors, T2DM, neurodegeneration, and/or hepatic steatosis, depending on dose and route of administration [17-25]. Therefore, better characterization of the non-neoplastic and degenerative effects of nitrosamines is warranted.

STZ, like other N-nitroso compounds, functions as: 1) an alkylating agent and potent mutagen [17]; 2) an inducer of DNA adducts leading to apoptosis [26]; 3) a mediator of unscheduled DNA synthesis, triggering cell death [17]; 4) an inducer of single-strand DNA breaks and stimulus for nitric oxide (NO) formation following breakdown of its nitrosamine group [19]; and 5) an enhancer of the xanthine oxidase system leading to increased production of superoxide anion, H_2_O_2_, and OH^- ^radicals [27]. Ultimately, STZ-induced injury and DNA damage promote mitochondrial dysfunction [19], ATP deficits [28], and cell death. The structural similarities between STZ and nitrosamines, including N-nitrosodiethylamine (NDEA) and N-nitrosodimethylamine (NDMA) [29], together with experimental evidence that high doses of STZ cause cancer while lower doses cause diabetes or AD-type neurodegeneration with cognitive impairment [18,19,25], led us to hypothesize that while high doses of nitrosamine exposures cause cancer, lower, sub-mutagenic doses may promote insulin resistance-mediated degenerative diseases.

There is growing evidence that obesity, T2DM, and cognitive impairment are inter-related based on findings that: 1) the risk of developing mild cognitive impairment (MCI), dementia, or AD is increased in individuals with T2DM [10,30] or obesity/dyslipidemic disorders [31]; 2) brain insulin resistance and insulin deficiency progress with severity of AD [32-35]; 3) experimental models of T2DM and/or obesity exhibit MCI [36,37] and neurodegeneration with brain insulin resistance [38,39]; 4) experimentally induced brain insulin resistance and insulin deficiency cause AD-type neurodegeneration and cognitive impairment [24,25,40-42]; 5) insulin sensitizer agents or intranasal insulin [43-49] can improve cognitive performance in experimental models of AD [22] or in humans with AD or MCI; and 6) similar molecular, biochemical, and mechanistic abnormalities in T2DM, NASH, and AD [10-14,16,50]. However, chronic high fat diet (HFD)-induced obesity that leads to NASH and T2DM is not sufficient to cause AD; instead, the associated neurodegeneration is relatively mild, despite brain insulin resistance [38,39]. Correspondingly, most individuals with T2DM do not develop AD, and the vast majority of individuals with AD are neither obese nor diabetic. Therefore, some additional co-factors must determine who among the at-risk population will likely develop AD. Herein, we examined the hypothesis that early sub-mutagenic exposure to NDEA and chronic HFD feeding additively or synergistically mediate the triad of major insulin resistances diseases, including T2DM, NASH, and AD-type neurodegeneration with cognitive impairment.

## Materials and methods

### Materials

Chow high fat (D12492-60% of calories) and low fat (D12450B-10% of calories) diets were commercially prepared by Research Diets, Inc. (New Brunswick, NJ). Rabbit, mouse, or goat generated monoclonal or polyclonal antibodies to ubiquitin, tau, phospho-tau (AT8-S199, S202, T205), glial fibrillary acidic protein (GFAP), 4-hydroxy-2-nonenal (HNE), choline acetyltransferase (ChAT), amyloid precursor protein amyloid-β peptide (Aβ PP-Aβ), β-actin, were purchased from Chemicon (Tecumsula, CA), CalBiochem (Carlsbad, CA), or Molecular Probes (Eugene, OR). All other polyclonal and monoclonal antibodies and immunodetection reagents were purchased from Abcam (Cambridge, MA), Vector Laboratories (Burlingame, CA), Upstate (Billerica, MA), Chemicon (Temecula, CA), or Molecular Probes (Eugene, OR). The insulin ultra-sensitive ELISA kit was obtained from ALPCO Diagnostics (Salem, NH). Histochoice fixative was purchased from Amresco, Inc (Solon, OH). Infinity Alanine aminotransaminase (ALT/GPT) assay kits were purchased from Fisher Diagnostics (Middletown, VA). Antibodies to tumor necrosis factor-α (TNF-α) and interleukin-1β (IL-1β) were purchased from Invitrogen (Carlsbad, CA). Antibodies to Akt, phospho-Akt (Thr308), GSK3β, and phospho-GSK-3β (Ser9) were purchased from Cell Signaling (Danvers, MA). The Amplex Red Cholesterol Assay Kit and Amplex UltraRed soluble fluorophore were purchased from Invitrogen (Carlsbad, CA). MaxiSorb 96-well plates used for ELISAs were from Nunc (Thermo Fisher Scientific; Rochester, NY). The TopCount NXT and ATP Lite assay kit were from Perkin-Elmer (Waltham, MA). Superblock-TBS, horseradish peroxidase conjugated antibodies, and SuperSignal Enhanced Chemiluminescence Reagent were from Pierce Chemical Co (Rockford, IL). QIAzol Lysis Reagent for RNA extraction and QuantiTect SYBR Green PCR Mix were obtained from Qiagen, Inc (Valencia, CA). The AMV 1^st ^Strand cDNA Synthesis kit was purchased from Roche Applied Science (Indianapolis, IN). [^125^I]-Labeled recombinant insulin and IGF-I polypeptides were purchased from GE Healthcare (Picataway, NJ), and unlabeled recombinant insulin and IGF-1 polypeptides were purchased from Bachem Americas, Inc. (Torrance, CA). The Serum Triglyceride Determination kit and synthetic oligonucleotides used in quantitative polymerase chain reaction (qPCR) assays were purchased from Sigma-Aldrich Co (St. Louis, MO). Fine chemicals were purchased from CalBiochem (Carlsbad, CA) or Sigma-Aldrich (St. Louis, MO).

### Experimental Model

Postnatal day 3 (P3) Long Evans rat pups (mean body weight 10 g) were given 3 alternate day intra-peritoneal (i.p.) injections of 20 μg NDEA or vehicle. Upon weaning, rats (N = 8-10 per group) were pair-fed for 6 weeks with high fat (HFD) or low fat (LFD) chow diets. The HFD supplied 60% of the kcal in fat (54% from lard, 6% from soybean oil), 20% in carbohydrates, and 20% in protein, whereas the LFD supplied 10% of the kcal in fat (4.4% from lard, 5.6% from soybean oil), 70% in carbohydrates, and 20% in protein. The use of young rats enabled us to compare results with previous observations in the STZ model [51]. Moreover, longitudinal studies of nuns revealed that neuro-cognitive deficits precede the onset of dementia by decades [52,53], suggesting that early life exposures may contribute to the pathogenesis of AD.

Rats were weighed weekly, and food consumption was monitored daily. At the end of the feeding period, the rats were then subjected to Morris Water Maze testing of spatial learning and memory [22,25]. Then, after an overnight fast (14 hours), rats were sacrificed by i.p. injection of pentobarbital (120 mg/kg), and blood, pancreas, liver, and brain (temporal lobes) were harvested. Blood or serum was used to measure glucose, insulin, cholesterol, triglyceride, free fatty acid, and alanine transaminase (ALT) as previously described [38,39]. The tissues were used for histological, biochemical, and molecular studies.

For histological studies, tissues were immersion fixed in Histochoice, embedded in paraffin, and sections (5-μm thick) were stained with Hematoxylin and Eosin (H&E-liver and pancreas) or Luxol fast blue, H&E (temporal lobes) and examined under code. For molecular and biochemical studies, liver and temporal lobe samples were snap-frozen in a dry ice-methanol bath and stored at -80°C [22,25,38,39]. The temporal lobes were studied because they: 1) require intact insulin/IGF signaling mechanisms to maintain structural and functional integrity [51,54]; 2) are severely damaged by i.c.-STZ mediated neurodegeneration [22,25]; and 3) are major targets of neurodegeneration in AD [51]. Our experimental protocol was approved by the Institutional Animal Care and Use Committee at Lifespan-Rhode Island Hospital, and conforms to the guidelines set by the National Institutes of Health.

### Morris Water Maze Testing

Morris Water Maze testing [55] of spatial learning and memory was performed on 4 consecutive days as previously described [22,25]. On the first day of testing, the rats were oriented to the water maze and educated about the location of the platform. On the 3 subsequent days of testing, the platform was submerged just below the surface, and rats were tested for learning and memory by measuring the latency period required to reach and recognize the platform. The rats were placed in the same quadrant of the water maze for every trial on Days 1 and 2, but on days 3 and 4, the start locations were randomized. Data from all 3 trials on each day were used to calculate latency area under the curve. Inter-group comparisons were made using the Kruskal-Wallis one-way ANOVA and Dunn's multiple comparison post-hoc test of significance.

### Lipid Assays

Lipid analyses were performed with serum samples and chloroform-methanol (2:1) extracted fresh frozen liver tissue homogenates [38]. Total lipid content was measured using a Nile Red fluorescence-based assay [56-58], and fluorescence intensity (Ex 485/Em 572) was measured in a SpectraMax M5 microplate reader (Molecular Devices Corp., Sunnyvale, CA). Triglycerides, cholesterol, and free fatty acids were measured with commercially available assay kits.

### Quantitative Reverse Transcriptase Polymerase Chain Reaction (qRT-PCR) Assays of Gene Expression

Total RNA was reverse transcribed with random primers, and the cDNA templates were PCR amplified with gene specific primer pairs [25] (Table 1). The amplified products were detected and analyzed in triplicate using the Mastercycler ep realplex instrument and software (Eppendorf AG, Hamburg, Germany) [38,59]. Relative mRNA abundance was calculated from the ng ratios of mRNA to 18S rRNA measured in the same samples, and those data were used for inter-group comparisons. Control studies included analysis of: 1) template-free reactions; 2) RNA that had not been reverse transcribed; 3) RNA samples pre-treated with DNAse I; 4) samples treated with RNAse A prior to the reverse transcriptase reaction; and 5) genomic DNA. All assays were performed in triplicate.

**Table 1:** Primer Pairs Used for Quantitative Reverse Transcriptase Polymerase Chain Reaction Assays

Primer	Direction	Sequence (5'→3')	Position (mRNA)	Amplicon Size (bp)
Insulin	For	TTC TAC ACA CCC AAG TCC CGT C	145	135

Insulin	Rev	ATC CAC AAT GCC ACG CTT CTG C	279	

Insulin Receptor	For	TGA CAA TGA GGA ATG TGG GGA C	875	129

Insulin Receptor	Rev	GGG CAA ACT TTC TGA CAA TGA CTG	1003	

IGF-I	For	GAC CAA GGG GCT TTT ACT TCA AC	65	127

IGF-I	Rev	TTT GTA GGC TTC AGC GGA GCA C	191	

IGF-I Receptor	For	GAA GTC TGC GGT GGT GAT AAA GG	2138	113

IGF-I Receptor	Rev	TCT GGG CAC AAA GAT GGA GTT G	2250	

IGF-II	For	CCA AGA AGA AAG GAA GGG GAC C	763	95

IGF-II	Rev	GGC GGC TAT TGT TGT TCA CAG C	857	

IGF-II Receptor	For	TTG CTA TTG ACC TTA GTC CCT TGG	1066	91

IGF-II Receptor	Rev	AGA GTG AGA CCT TTG TGT CCC CAC	1156	

IRS-1	For	GAT ACC GAT GGC TTC TCA GAC G	604	134

IRS-1	Rev	TCG TTC TCA TAA TAC TCC AGG CG	737	

IRS-2	For	CAA CAT TGA CTT TGG TGA AGG GG	255	109

IRS-2	Rev	TGA AGC AGG ACT ACT GGC TGA GAG	263	

IRS-4	For	ACC TGA AGA TAA GGG GTC GTC TGC	2409	132

IRS-4	Rev	TGT GTG GGG TTT AGT GGT CTG G	2540	

ChAT	For	TCA CAG ATG CGT TTC ACA ACT ACC	478	106

ChAT	Rev	TGG GAC ACA ACA GCA ACC TTG	583	

AChE	For	TTC TCC CAC ACC TGT CCT CAT C	420	123

AChE	Rev	TTC ATA GAT ACC AAC ACG GTT CCC	542	

APP	For	GCA GAA TGG AAA ATG GGA GTC AG	278	199

APP	Rev	AAT CAC GAT GTG GGT GTG CGT C	476	

Tau	For	CGC CAG GAG TTT GAC ACA ATG	244	65

Tau	Rev	CCT TCT TGG TCT TGG AGC ATA GTG	308	

SPTLC-1	For	CTAACCTTGGGCAAATCGAA	2581	96

SPTLC-1	Rev	TGAGCAGGGAGAAGGGACTA	2676	

SPTLC-2	For	GGA CAG TGT GTG GCC TTT CT	1823	50

SPTLC-2	Rev	TCA CTG AAG TGT GGC TCC TG	1872	

CERS-1	For	TGC GTG AAC TGG AAG ACT TG	947	98

CERS-1	Rev	CTT CAC CAG GCC ATT CCT TA	1044	

CERS-2	For	CTC TGC TTC TCC TGG TTT GC	698	82

CERS-2	Rev	CCA GCA GGT AGT CGG AAG AG	779	

CERS-4	For	CGA GGC AGT TTC TGA AGG TC	1240	72

CERS-4	Rev	CCA TTG GTA ATG GCT GCT CT	1311	

CERS-5	For	GAC AGT CCC ATC CTC TGC AT	1254	92

CERS-5	Rev	GAG GTT GTT CGT GTG TGT GG	1345	

UGCG	For	GAT GCT TGC TGT TCA CTC CA	2682	67

UGCG	Rev	GCT GAG ATG GAA GCC ATA GG	2748	

SMPD-1	For	CAG TTC TTT GGC CAC ACT CA	1443	65

SMPD-1	Rev	CGG CTC AGA GTT TCC TCA TC	1507	

SMPD-3	For	TCT GCT GCC AAT GTT GTC TC	2704	98

SMPD-3	Rev	CCG AGC AAG GAG TCT AGG TG	2801	

*Abbreviations: IGF = insulin-like growth factor; IRS = insulin receptor substrate; ChAT = choline acetyltransferase; AChE = acetylcholinesterase; APP = amyloid precursor protein; SPTLC = serine palmitoyltransferase; CERS = ceramide synthase; UGCG = UDP-glucose ceramide glucosyltransferase; SMPD = sphingomyelin phosphodiesterase; bp = base pair.

### Enzyme-Linked Immunosorbant Assay (ELISA)

Tissues were homogenized in radioimmunoprecipitation assay buffer containing protease and phosphatase inhibitors [39]. Direct ELISAs were performed in 96-well Maxisorb plates [60]. In brief, proteins (40 ng/100 μl) adsorbed to well bottoms by over night incubation at 4°C, were blocked with 1% BSA in phosphate buffered saline (PBS), and then incubated with primary antibody (0.2-1.0 μg/ml) for 1 hour at 37°C. Immunoreactivity was detected with horseradish peroxidase (HRP)-conjugated secondary antibody (1:10000) and UltraRed soluble fluorophore [60].

For capture ELISAs used to measure pro-inflammatory cytokines, capture antibodies (1 μg/ml) were adsorbed to the well bottoms overnight, and after blocking for 3 hours at room temperature with 1% BSA prepared in PBS + 0.05% Tween-20, 50 ng of liver homogenate protein were added to each well in a 100 μl volume, and incubated for 2 hours at room temperature. Reactions were then incubated with biotinylated detection antibodies (0.2 μg/ml for IL-1β and TNFα, and 0.5 μg/ml for IL-6) for 2 hrs at room temperature. Immunoreactivity was revealed with HRP-conjugated Streptavidin and Amplex UltraRed. Fluorescence was measured (Ex 568/Em 581) in a SpectraMax M5 microplate reader. Binding specificity was determined from parallel negative control incubations with the primary or secondary antibody omitted. Immunoreactivity was normalized to protein content in parallel wells as determined with the NanoOrange Protein Quantification Kit.

### Receptor Binding Assays

Competitive equilibrium binding studies were used to measure effects of NDEA and HFD feeding on insulin and IGF-I receptor binding in liver and temporal lobe [39,61]. For total binding, NP-40 lysis buffer homogenates were incubated with 50 nCi/ml of [^125^I] (2000 Ci/mmol; 50 pM) insulin or IGF-I in binding buffer (100 mM HEPES (4-(2-hydroxyethyl)-1-piperazine-ethanesulfonic acid), pH 8.0, 118 mM NaCl, 1.2 mM MgSO4, 8.8 mM dextrose, 5 mM KCl, 1% bovine serum albumin). For non-specific binding, identical reactions were prepared with the addition of 0.1 μM unlabeled (cold) ligand. After 16-hours incubation at 4°C, reactions were vacuum harvested (Corning, Lowell, MA) onto 96-well GF/C filter plates that were pre-soaked in 0.33% polyethyleneimine. The filters were vacuum washed with 50 mM HEPES, pH 7.4, 500 mM NaCl, and 0.1% BSA. [125I]- bound insulin or IGF-I was measured in a TopCount. Specific binding was calculated by subtracting non-specifically bound from the total bound isotope [39].

### Statistical Analysis

Data depicted in the graphs and tables represent the means ± S.E.M.'s for 8-10 samples per group. Inter-group comparisons were made using two-way ANOVA and post-hoc Bonferroni's multiple comparison test of significance. Statistical analyses were performed using the GraphPad Prism 5 software (GraphPad Software, Inc., San Diego, CA). Significant P-values are shown in the graphs or included in the tables.

## Results

### Effects of NDEA and HFD on Serum Biomarkers of T2DM, Dyslipidemia, Body Weight, and Brain Weight

**Figure 1:**
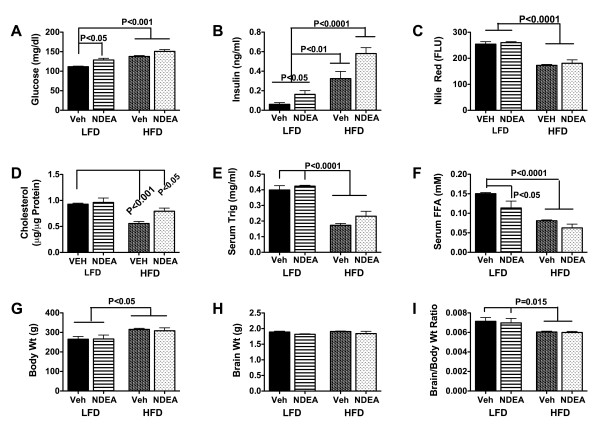
**NDEA and HFD Feeding Cause T2DM Without Hyper-lipidemia: Long Evans rats were treated with 3 i.p. injections of vehicle or NDEA (N = 12/group) on alternate days beginning on postnatal day 3 (P3)**. From P21 (weaning), rats were fed with high fat (60% of calories) or low fat (5% of calories) diets for 6 weeks. Serum harvested at the time of sacrifice and after an over-night fast was used to measure (A) glucose, (B) insulin, (C) Nile Red fluorescence (neutral lipids), (D) cholesterol, (E) triglycerides, and (F) free fatty acids. In addition, the mean (G) body weights, (H) brain weights, and (I) calculated brain weight/body weight ratios at the time of sacrifice are shown. Graphs depict the mean ± S.E.M. results for each group. Data were analyzed using repeated measures ANOVA and the post-hoc Dunn's test. Significant inter-group differences are indicated by the P-values over the bars.

Fasting blood glucose and serum insulin concentrations were significantly higher in NDEA-treated rats, with or without HFD-feeding, relative to LFD fed, vehicle treated controls, and the highest mean blood glucose and serum insulin levels were detected in the HFD+NDEA group (Figs 1A-B). Serum neutral lipid (Nile Red assay) (Fig. 1C), cholesterol (Fig. 1D), triglyceride (Fig. 1E), and free fatty acid (Fig. 1F) levels were either unchanged or significantly reduced in the NDEA, HFD, and HFD+NDEA treated groups relative to control, and the lowest mean serum lipid levels were measured in the HFD ± NDEA treated groups. Therefore, hyperglycemia and hyper-insulinemia were features of chronic HFD feeding, and these indices of peripheral insulin resistance were worsened by prior exposure to low-dose NDEA. In addition, early NDEA exposure alone was sufficient to cause mild but significant peripheral insulin resistance. In contrast, neither the NDEA exposure nor the chronic HFD feeding caused hyperlipidemia, indicating that seemingly favorable serum lipid profiles can exist in the context of peripheral insulin resistance or T2DM. These findings are similar to those in a previous report in which the experimental model was generated with much higher doses of NDEA than used herein [62]. One possible interpretation of these seemingly paradoxical results is that homeostasis may have shifted toward increased storage of lipids/triglycerides in adipose tissue, skeletal muscle, and/or liver. Although initial mean body weights were similar among the groups (data not shown), at the time of sacrifice, HFD ± NDEA treated rats were significantly heavier than LFD ± NDEA treated rats (Fig. 1G). The mean brain weights did not differ significantly among the groups (Fig. 1H), but the brain/body weight ratios were significantly reduced in HFD ± NDEA relative to the LFD ± NDEA treated rats (Fig. 1I), in part due to their larger body masses.

### NDEA and HFD-Induced Islet Hypertrophy and Steatohepatitis (Table 2 and Fig. 2)

**Figure 2:**
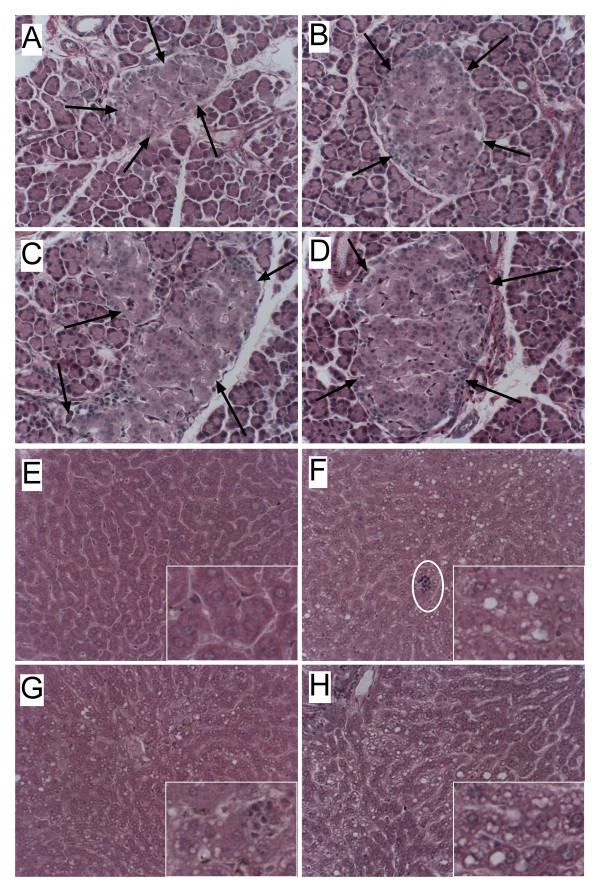
**NDEA Exposure and Chronic HFD Feeding Induce Pancreatic Islet hypertrophy and Steatohepatitis**. Long Evans rats were treated with sub-mutagenic doses of NDEA or vehicle (Veh) and then fed with high (HFD) or low fat (LFD) containing chow for 6 weeks (see legend to Figure 1). Pancreas and liver tissues were immersion fixed and embedded in paraffin. Histological sections were stained with H&E. Photomicrographs depict (A-D) pancreas or (E-H) liver from (A, E) LFD+Veh control, (B, F) HFD+Veh, (C, G) LFD+NDEA, and (D, H) HFD+NDEA treated rats. Note enlarged islets in Panels B, C, and D relative to A (arrows). (F) Chronic HFD feeding increased hepatic macrosteatosis (mainly large clear vacuoles; inset) and foci of lymph-mononuclear cell inflammation (encircled) relative to (E) control. Treatment with (G) NDEA resulted in disruption of the regular chord-like arrangement of hepatocytes, increased macro- (large) and microvesicular (small) steatosis, and increased foci of inflammation, and necrosis (inset). (H) NDEA+HFD exposure further disrupted the hepatic chord architecture, and increased the density of lipid vacuoles (inset), and foci of inflammation and necrosis. A-D, Original magnification 400×; E-H Original magnifications, Panels-80×; insets-650×.

**Table 2:** Assays of Liver Injury or Hepatic Steatosis

Assay^§^	LFD+VEH	LFD+NDEA	HFD+VEH	HFD+NDEA	F-Ratio	P-Value
Serum ALT (U/L)	17.3 ± 0.9	23.8 ± 3.9	30.6 ± 1.8**	33.5 ± 2.1**	10.08	< 0.0001
Nile Red (RFU/μg)	111.0 ± 2.6	128.3 ± 15.0	122.7 ± 6.1	147.9 ± 6.1*	2.77	0.06
Cholesterol (ng/mg)	360.5 ± 20.2	340.1 ± 33.9	435.4 ± 21.9	458.4 ± 19.7*	5.02	0.011
Triglycerides (μg/mg)	11.2 ± 1.5	10.8 ± 2.0	17.1 ± 0.8*	21.0 ± 1.4**	10.3	0.0003

§ALT = alanine transaminase, Nile Red, cholesterol, and tryglyceride levels were measured in liver tissue. RFU/μg = relative fluorescence units; Nile red, cholesterol and triglyceride measurements are relative to liver. Inter-group comparisons were made using ANOVA tests. For all measurements, df = 3/36. Significant differences relative to the LFD control group were demonstrated using the Dunnett's post-hoc multiple comparison test (*P < 0.05; **P < 0.01; ***P < 0.001).

Corresponding with the significantly increased blood glucose and insulin levels, the HFD (Fig. 2B), NDEA (Fig. 2C), and HFD+NDEA (Fig. 2D) treated rats had conspicuously hypertrophied pancreatic islets relative to LFD+Vehicle (Veh) treated controls (Fig. 2A). Islet hypertrophy was not associated with inflammation, necrosis, or histopathologic abnormalities in the exocrine pancreas. Livers of LFD+Veh treated rats had regular chord-like architectures with minimal steatosis and no inflammation (Fig. 2E). NDEA, HFD, and HFD+NDEA treated rats all had mixed patterns of macrovesicular and microvesicular hepatic steatosis (Figs. 2F-2H) with scattered foci of lymphomononuclear cell inflammation (Fig. 2F and inset). In addition, livers of NDEA ± HFD treated rats had conspicuous disorganization of the hepatic chord architecture with scattered foci of hepatocellular necrosis (Figs. 2G-H). A distinguishing feature of the NDEA+HFD exposed livers was the strikingly greater prominence of hepatocellular disorganization and steatosis relative to the other groups (Fig. 2H). None of the livers showed evidence of malignant transformation, i.e. tumors, nodules, or foci of anaplasia. The NDEA ± HFD abnormalities in liver correspond with the effects of STZ treatment [63-65], and are highly consistent with features of NASH in humans [1,12,66]. Biochemical assays confirmed that chronic HFD feeding ± NDEA treatment caused mild liver injury and steatosis as demonstrated by the significantly increased serum levels of ALT, and hepatic triglyceride content (Table 2). The finding that the NDEA+HFD group had the highest mean levels of serum ALT, and hepatic Nile Red, cholesterol, and triglycerides suggests that the effects of the NDEA plus HFD feeding were additive with respect to liver injury.

### Morris Water Maze Performance (Figure 3)

**Figure 3:**
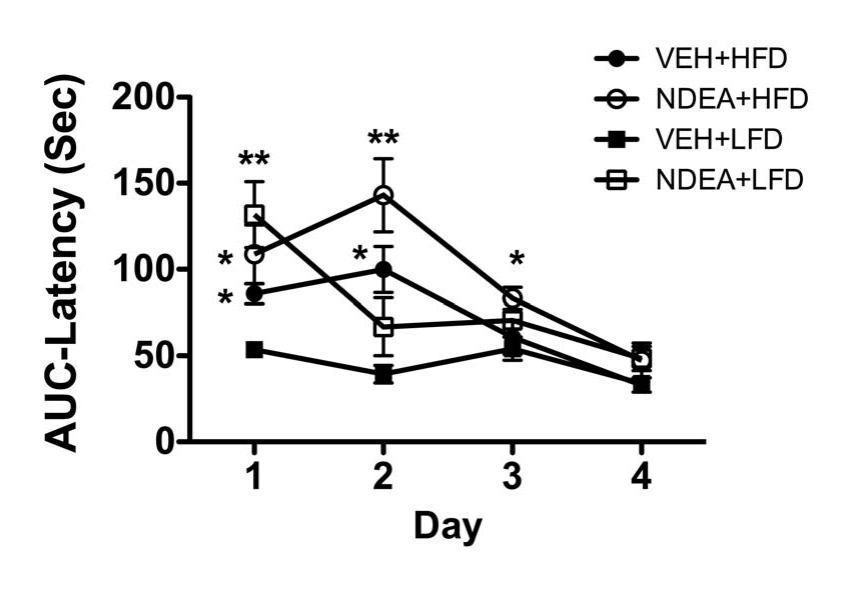
**NDEA Treatment and Chronic HFD Feeding Impair Spatial Learning and Memory: Long Evans rats were treated with NDEA or vehicle (VEH) by i.p. injection (N = 12/group), and then chronically fed with high fat (HFD) or low fat (LFD) containing chow for 6 weeks (see Figure Legend 1)**. Rats were subjected to Morris Water Maze testing on 4 consecutive days. On Day 1, the platform was visible, but on Days 2-4, the platform was submerged, and on Days 3-4, the water entry quadrant was randomized. On each testing day, rats were given 3 trials, with a maximum of 120 seconds allowed to land on the platform, beyond which they were guided. Area under curve (AUC) was calculated for each series of trials each day. Graphs depict the mean ± S.E.M. AUC latencies for each group on each day of testing. Data were analyzed using the Kruskal-Wallis one-way ANOVA and Dunn's multiple comparison post-hoc test for significance. Significant P-values relative to control (VEH+LFD) are indicated by asterisks (*P < 0.05; **P < 0.01).

On Day 1 of testing (acquisition phase), NDEA treatment, with or without chronic HFD feeding, significantly impaired performance. On Day 2, HFD ± NDEA treated rats performed significantly worse than LFD+Veh treated controls. On Day 3, the NDEA+HFD group still performed significantly worse than control, and although the NDEA+LFD group had a longer mean latency for locating the hidden platform, the difference from control was not statistically significant. On Day 4, the mean latencies in the NDEA-treated groups remained slightly higher than Veh-treated LFD and HFD fed rats, but the inter-group differences did not reach statistical significance. In essence, although the mean (area under curve) latencies for locating and landing on the platform declined over time in all groups, NDEA treatment alone impaired performance during the acquisition phase, while combined NDEA+HFD exposures significantly impaired both learning and memory.

### Neuropathology of NDEA Exposure

**Figure 4:**
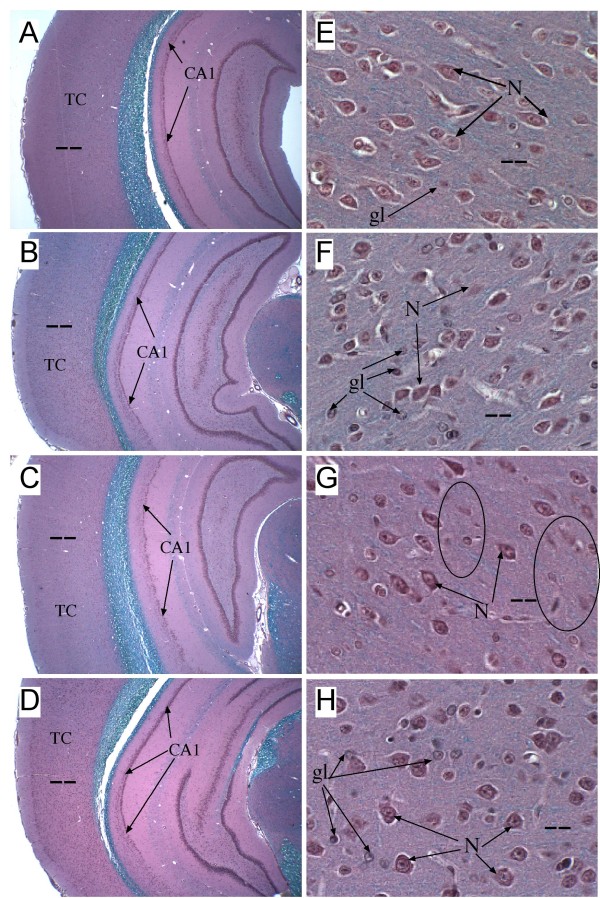
**Hippocampus and Temporal Lobe Degeneration Following NDEA-Exposure and/or HFD Feeding: Long Evans rats treated with NDEA or vehicle (Veh) by i.p. injection (N = 12/group), and then chronically fed with high fat (HFD) or low fat (LFD) containing chow for 6 weeks (see Figure Legend 1)**. Brains were immersion fixed and embedded in paraffin. Histological sections were stained with Luxol fast blue, H&E. Blue staining corresponds to myelinated fibers and tracts. Photomicrographs depict (A-D) low magnification images of temporal cortex (TC) and hippocampal formation and (E-H) high magnification images of the temporal cortex. Note the thinning/reduced cell density within the CA1 region (Segment 1 of Ammon's Horn; arrows) of the hippocampal formation and slight thinning of the TC in brains of (B) Veh+HFD, (C) NDEA+LFD, and (D) NDEA+HFD treated rats relative to control (A) Veh+LFD). Higher magnification images of TC show (E) abundant pyramidal neurons (N) with regular organization and scattered glia (gl) in LFD+Veh treated control brains, (F) neuronal atrophy and apoptosis (N; arrows) and increased glia (gl) in Veh+HFD treated rats, and (G, H) conspicuous neuronal loss with small clusters of apoptotic cells (encircled) in NDEA ± HFD-treated rats. In addition, as observed in the HFD-fed rat brains, glial cells were conspicuously increased relative to neurons in the (H) HFD+NDEA treated temporal cortex. Original magnifications: A-D, 40×; E-H, 400×. (Scale bar A-D, 200 microns; E-H 25 microns).

LFD+Veh treated control Ammon's horn (CA1-CA4 regions) of the hippocampus (Fig. 4A) and the temporal cortex (Fig. 4E) were richly populated by neurons with excellent preservation of the architecture (Fig. 4A). Chronic HFD feeding, NDEA-treatment, and NDEA+HFD exposure resulted in thinning of CA1 (Figs. 4B-4D), and atrophy of the temporal cortex with loss of neurons manifested by increased inter-neuronal spacing and on-going apoptosis (Figs. 4F-H). In addition, glial cells were conspicuously increased in density in the temporal cortex of NDEA+HFD treated rats (Fig. 4H).

### Limited NDEA-Exposure and Chronic HFD Feeding Cause Sustained Abnormalities in Expression of AD-Associated Genes

**Figure 5:**
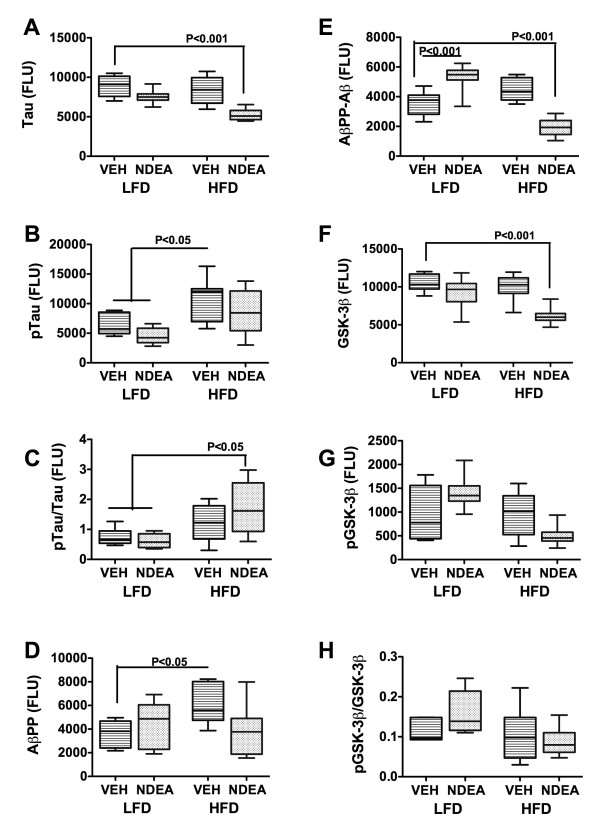
**Effect of NDEA Exposure and Chronic HFD Feeding on Molecular Indices of Neurodegeneration**. Long Evans rats were treated with NDEA or vehicle (VEH) by i.p. injection (N = 12/group), and then chronically fed with high fat (HFD) or low fat (LFD) containing chow for 6 weeks (see Figure Legend 1). Temporal lobe protein homogenates were used to measure (A) Tau; (B) phospho (p)-Tau; (D) AβPP, (E) AβPP-Aβ, (F) GSK-3β, or (G) phospho (p)-GSK-3β by direct binding ELISA. In addition, the ratios of (C) pTau/Tau and (H) pGSK-3β/GSK-3β were calculated. Immunoreactivity was detected with HRP-conjugated secondary antibody and Amplex Red soluble fluorophor. Fluorescence light units (FLU) were measured (Ex 579 nm/Em 595 nm) in a Spectromax M5, and results were normalized to protein content in the wells. Box plots depict mean ± S.E.M of results. Inter-group comparisons were made using ANOVA with the post-hoc Bonferroni test of significance. Significant P-values are indicated within the panels.

**Figure 6:**
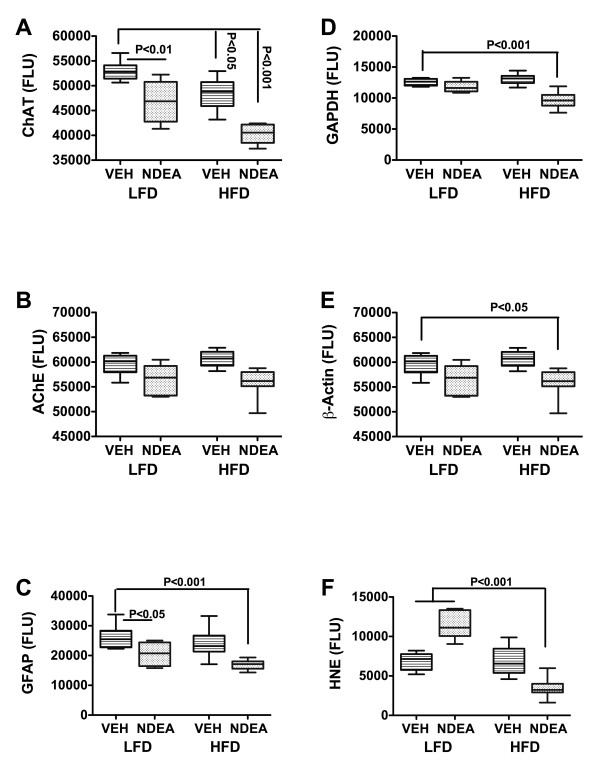
**Effect of NDEA Exposure and Chronic HFD feeding on Molecular Indices of Oxidative Stress and Neurodegeneration**. Long Evans rats were treated with NDEA or vehicle (VEH) by i.p. injection (N = 12/group), and then chronically fed with high fat (HFD) or low fat (LFD) chow for 6 weeks (see Figure Legend 1). Temporal lobe protein homogenates were used to measure (A) choline acetyltransferase (ChAT), (B) acetylcholinesterase (AChE), (C) GFAP, (D) GAPDH, (E) β-Actin, or (F) 4-hydroxynonenal (HNE) by direct binding ELISA. Immunoreactivity was detected with HRP-conjugated secondary antibody and Amplex Red soluble fluorophor. Fluorescence light units (FLU) were measured (Ex 579 nm/Em 595 nm) in a Spectromax M5, and results were normalized to sample protein content in the wells. Box plots depict mean ± S.E.M of results. Inter-group comparisons were made using ANOVA with the post-hoc Bonferroni test of significance. Significant P-values are indicated within the panels.

To assess long-term effects of NDEA ± HFD on genes and proteins that are aberrantly expressed in AD, we measured amyloid-β-precursor protein (AβPP), Tau, choline acetyltransferase (ChAT), acetylcholinesterase (AChE), interleukin 1β (IL-1β), IL-6, and tumor necrosis factor-alpha (TNF-α) mRNA levels by qRT-PCR analysis (Table 3), and Tau, phospho-Tau, AβPP, AβPP-Aβ, ChAT, AChE, glyceraldehyde-3-phosphate dehydrogenase (GAPDH), β-Actin, GFAP, HNE, glycogen synthase kinase 3β (GSK-3β), and pGSK-3β immunoreactivity by ELISA (Figs. 5 and 6). The qRT-PCR studies demonstrated that chronic HFD feeding alone reduced AβPP gene expression (P < 0.05), while NDEA ± HFD significantly reduced Tau (P < 0001), ChAT (P < 0.001), and IL-1β (P < 0.05) mRNA expression relative to LFD+Veh treated controls (Table 3). Although AβPP mRNA expression was also reduced in the NDEA+HFD group, the difference from control did not reach statistical significance. The mean mRNA levels of AChE, IL-6 and TNF-α were not significantly altered in the HFD, NDEA, or NDEA+HFD groups relative to control.

**Table 3:** Effects of High Fat Diet and NDEA Exposure on Biomarkers of AD and Neuro-inflammation in Temporal Lobe

	LFD	LFD+NDEA	HFD	HFD+NDEA	F-Ratio	P-Value
AβPP	4.392 ± 0.423	3.966 ± 0.458	3.026* ± 0.215	3.228 ± 0.299	3.099	0.039
Tau	3.380 ± 0.272	2.689 ± 0.255	3.310 ± 0.270	1.938*** ± 0.209	7.023	0.001
ChAT	0.170 ± 0.016	0.147 ± 0.026	0.164 ± 0.014	0.115^§ ^± 0.007		0.001
AChE	0.824 ± 0.081	1.080 ± 0.137	0.747 ± 0.099	0.795 ± 0.048		
IL-1β	0.047 ± 0.004	0.047 ± 0.010	0.052 ± 0.005	0.023* ± 0.003	4.627	0.008
IL6	0.052 ± 0.006	0.045 ± 0.006	0.050 ± 0.005	0.034 ± 0.005		
TNF-α	0.022 ± 0.002	0.035 ± 0.005	0.034 ± 0.005	0.036 ± 0.004		

Gene expression was measured by qRT-PCR with results normalized to 18S rRNA (see Methods). Values correspond to mRNA/18S × 10^-6^. Inter-group comparisons were made using ANOVA tests. For all measurements, df = 3/36. Significant differences relative to the LFD control group were demonstrated using the Dunnett's post-hoc multiple comparison test (*P < 0.05; **P < 0.01; ***P < 0.001). For ChAT, the ANOVA test was not significant, but the mRNA levels were significantly different from control in the HFD+NDEA treated group by T-test analysis.

ELISA results demonstrated that NDEA treatment alone significantly increased AβPP-Aβ (P < 0.001) and HNE (P < 0.001), and reduced ChAT (P < 0.01) and GFAP (P < 0.05) immunoreactivity relative to control (Figs. 5 and 6). Chronic HFD feeding significantly increased pTau (P < 0.05) and Aβ PP (P < 0.05), and reduced the mean levels of Chat (P < 0.05) relative to control (Figs. 5 and 6). The combined NDEA+HFD exposures significantly increased the mean ratio of pTau/Tau, indicating relatively higher tau phosphorylation, and reduced the mean levels of Tau (P < 0.001), AβPP-Aβ (P < 0.001), GSK-3β (P < 0.001), ChAT (P < 0.001), GAPDH (P < 0.001), GFAP (P < 0.001), β-actin (P < 0.05), and HNE (P < 0.001) relative to control (Figs. 5 and 6). AChE immunoreactivity was not significantly altered by NDEA and/or HFD feeding. Although GSK-3β immunoreactivity was reduced by NDEA+HFD exposure, the relative preservation of the pGSK-3β/GSK-3β ratio indicates that constitutive GSK-3β activity in brain was not significantly altered by these treatments. Therefore, the increased pTau/Tau ratio measured in the same samples may have been mediated by kinases other than GSK-3β. The additive effects of HFD plus NDEA exposure were demonstrated by the fact that the greater reductions in ChAT, tau, β-actin, GFAP, and GAPDH, and increases in the mean levels of pTau or pTau/Tau ratio compared with the effects of either treatment/exposure alone.

### Limited NDEA-Exposure and Chronic HFD Feeding Cause Sustained Impairments in Insulin/IGF Signaling Mechanisms in the Brain

QRT-PCR analysis demonstrated significantly increased levels of insulin (P < 0.05), IGF-1 (P < 001) and IRS-4 (P < 0.001) in rats exposed to NDEA only, increased insulin (P < 0.01) and IGF-2 receptor (P < 0.05), and decreased IGF-1, IGF-1 receptor (P < 0.05), and IRS-1 in rats chronically fed with the HFD, and reduced IGF-1 (P < 0.05), insulin receptor (P < 0.05), IGF-1 receptor (P < 0.05), and IRS-1 (P < 0.001) in rats treated with NDEA+HFD relative to LFD fed controls (Table 4). Moreover, insulin, IGF-1, insulin receptor, IGF-2 receptor, IRS-1, IRS-2, and IRS-4 mRNA levels were lowest in the NDEA+HFD treated group. These impairments in the expression of genes needed for proper insulin and IGF signaling are reminiscent of those detected in human brains with AD [34,51,67].

**Table 4:** Effects of High Fat Diet and NDEA Exposure on Biomarkers of Insulin and IGF Resistance in Temporal Lobe

	LFD	LFD+NDEA	HFD	HFD+NDEA	F-Ratio	P-Value
Insulin	0.093 ± 0.011	0.155* ± 0.015	0.181** ± 0.028	0.069 ± 0.008	9.1450	< 0.0001
IGF-1	0.170 ± 0.012	0.253** ± 0.029	0.101* ± 0.015	0.101* ± 0.007	16.4000	< 0.0001
IGF-2	2.336 ± 0.285	2.414 ± 0.675	1.718 ± 0.219	2.004 ± 0.179	19.3400	< 0.0001
Insulin R	1.060 ± 0.129	0.977 ± 0.070	0.997 ± 0.117	0.634* ± 0.073	3.6360	0.0210
IGF-1R	0.814 ± 0.111	1.027 ± 0.098	0.482* ± 0.047	0.485* ± 0.059	10.3700	< 0.0001
IGF-2R	0.649 ± 0.049	0.721 ± 0.107	0.893* ± 0.051	0.540 ± 0.053	4.5660	0.0080
IRS-1	0.393 ± 0.031	0.439 ± 0.040	0.244** ± 0.013	0.185*** ± 0.016	19.3400	< 0.0001
IRS-2	1.678 ± 0.211	2.652 ± 0.406	1.127 ± 0.104	1.300 ± 0.166		
IRS-4	0.029 ± 0.005	0.153*** ± 0.030	0.049 ± 0.007	0.025 ± 0.004	15.0000	< 0.0001

Gene expression was measured by qRT-PCR with results normalized to 18S rRNA (see Methods). Values correspond to mRNA/18S × 10^-6^. Inter-group comparisons were made using ANOVA tests. For all measurements, df = 3/36. Significant differences relative to the LFD control group were demonstrated using the Dunnett's post-hoc multiple comparison test (*P < 0.05; **P < 0.01; ***P < 0.001).

### NDEA and Chronic HFD Feeding Cause Hepatic and Brain Insulin and/or IGF-1 Resistance

**Figure 7:**
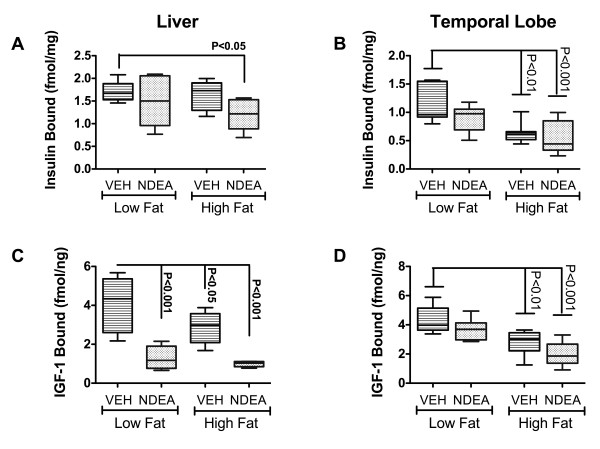
**NDEA Exposure and Chronic HFD Feeding Impair Insulin and IGF-1 Receptor Binding in Liver and Brain**. Long Evans rats were treated with NDEA or vehicle (VEH) by i.p. injection (N = 12/group), and then chronically fed with high fat (HFD) or low fat (LFD) containing chow for 6 weeks (see Figure Legend 1). Competitive equilibrium binding studies were used to measure specific insulin and IGF-1 binding to their corresponding receptors. Reactions were incubated with 50 nCi/ml of [^125^I] (2000 Ci/mmol; 50 pM) insulin or IGF-I in binding buffer in the presence or absence of 0.1 μM unlabeled ligand. Radiolabeled bound ligand was harvested onto 96-well GF/C filter plates and measured in a TopCount. Specific binding was calculated by subtracting non-specifically bound from the total bound isotope. Box plots depict mean ± S.D. of specific binding for (A, B) insulin and (C, D) IGF-I in (A, C) liver and (B, D) temporal lobe. Inter-group comparisons were made using ANOVA with the post-hoc Bonferroni test of significance. Significant P-values are indicated within the panels.

NDEA exposure significantly reduced IGF-1 receptor binding in liver (P < 0.001). Chronic HFD feeding significantly reduced IGF-1 receptor binding in liver (P < 0.05), and both IGF-1 (P < 0.01) and insulin (P < 0.01) receptor binding in brain. Combined NDEA+HFD exposures significantly reduced both insulin receptor binding in liver (P < 0.5) and brain (P < 0.001), and IGF-1 receptor binding in liver (P < 0.001) and brain (P < 0.001) (Fig. 7). In essence, insulin receptor binding was mainly associated with chronic HFD feeding, with or without antecedent NDEA exposure. However, the inhibitory effects of HFD feeding and NDEA exposure appeared to be additive with respect to insulin receptor binding in liver, and IGF-1 receptor binding in brain. These results suggest that the NDEA exposure and chronic HFD-feeding differentially mediate insulin and/or IGF resistance in liver and brain, indicating that a given exposure can adversely affect organ function in different ways and to dissimilar degrees.

### NDEA-Mediated Increases in Pro-Ceramide Gene Expression

**Figure 8:**
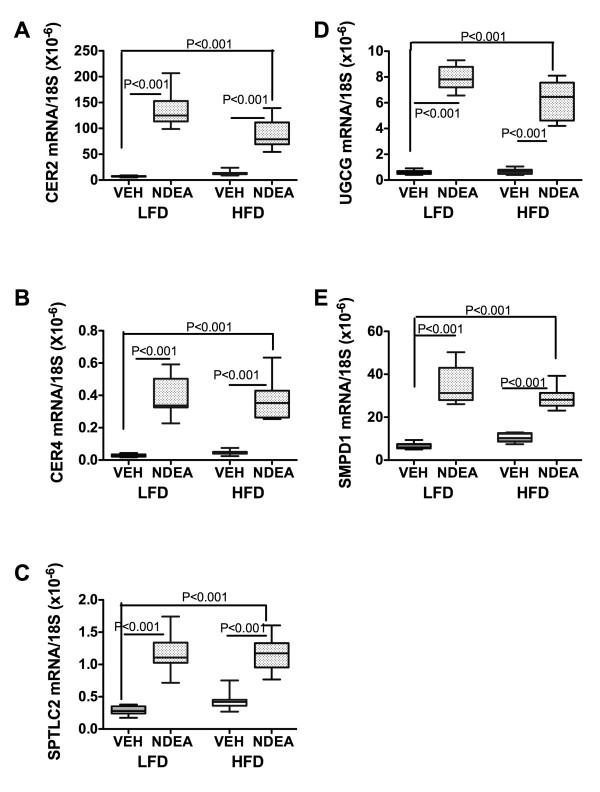
**Effect of NDEA exposure and chronic HFD feeding on pro-ceramide gene expression in liver**. Long Evans rats treated with NDEA or vehicle (VEH) by i.p. injection (N = 8/group), and then chronically fed with high fat (HFD) or low fat (LFD) containing chow for 6 weeks (see Figure Legend 1). Total RNA extracted from liver tissue was reverse transcribed using random oligodeoxynucleotide primers, and the resulting cDNA templates were used in qRT-PCR assays to measure (A) Ceramide synthase (CER) 2, (B) CER4, (C) Serine palmitoyltransferase 2 (SPTLC2), (D) UDP-glucose ceramide glycoysltransferase (UGCG), or (E) sphingomyelin phosphodiesterase 1 (SMPD1). The mRNA levels were normalized to 18S rRNA measured in the same templates. Graphs depict the mean ± S.E.M. levels of gene expression. Inter-group comparisons were made using ANOVA with the post-hoc Bonferroni test of significance. Significant P-values are indicated within the panels.

We investigated the potential role of pro-ceramide genes as mediators of NDEA ± HFD associated neurodegeneration because ceramides: 1) can be generated in brain and liver [68-71]; 2) cause cytotoxicity and insulin resistance [71,72]; and 3) are increased in brains with neurodegeneration [68,73-75]. Moreover, recent studies showed that increased ceramide gene expression in liver correlates with neurodegeneration in the context of obesity with T2DM and NASH [38], and that in vitro ceramide exposure causes neurodegeneration [72]. To assess the potential role of ceramides and their sources in relation to neurodegeneration and insulin/IGF-1 resistance, we measured mRNA levels of ceramide synthases (CER), UDP glucose ceramide glycosyltransferase (UGCG), serine palmitoyltransferase (SPTLC), and sphingomyelin phosphodiesterases (SMPD) in liver and temporal lobe, due to their demonstrated relevance to neurodegeneration [38,72]. The qRT-PCR analyses revealed significantly higher mean levels of all 5 genes in livers of NDEA ± HFD-treated relative to LFD+Veh or HFD+Veh treated rats (Fig. 8). However, there was no additional burden produced by chronic HFD, indicating that the NDEA exposure was sufficient to significantly alter pro-ceramide gene expression in the direction of increased ceramide production through both biosynthesis and degradation pathways. With regard to the temporal lobe, the effects of HFD and NDEA treatment were more varied compared with liver. NDEA treatment, with or without HFD feeding, increased expression of CER4, UGCG and SMPD3, but decreased CER2, CER5, and SPTLC1 mRNA levels (Table 5). In addition, HFD feeding alone significantly increased SMPD1 and decreased SPTLC1 expression relative to control. Therefore, both NDEA and HFD feeding contributed to increased ceramide generation in the brain, although some enzymes involved in ceramide biosynthesis were down-regulated, possibly as a protective/compensatory mechanism to modulate its accumulation.

**Table 5:** Effects of High Fat Diet and NDEA Exposure on Expression of Pro-Ceramide Genes in Temporal Lobe

	LFD	LFD+NDEA	HFD	HFD+NDEA	F-Ratio	P-Value
CER1	6.710	6.055	6.794	7.381		
	0.651	0.685	0.984	0.996		
CER2	19.230	4.858***	16.260	4.733**	18.780	< 0.0001
	2.017	0.752	2.509	0.495		
CER4	2.352	3.620*	2.770	4.608***	11.030	< 0.0001
	0.139	0.147	0.249	0.495		
CER5	18.370	4.898***	21.120	5.277***	18.540	< 0.0001
	2.517	0.295	2.707	0.576		
UGCG	13.670	29.960***	15.190	30.590***	15.330	< 0.0001
	0.929	3.605	1.667	2.827		
SMPD1	7.941	9.797	15.520***	9.490	6.919	0.001
	0.766	1.435	1.841	0.859		
SMPD3	4.405	28.910***	2.672	16.170**	21.170	< 0.0001
	0.396	5.078	0.325	0.603		
SPTLC1	0.066	0.038*	0.029**	0.042*	5.102	0.005
	0.010	0.008	0.004	0.006		
SPTLC2	1.728	1.107*	1.939	1.646	3.845	0.019
	0.190	0.207	0.173	0.115		

Gene expression was measured by qRT-PCR with results normalized to 18S rRNA (see Methods). Values correspond to mRNA/18S × 10^-6^. Inter-group comparisons were made using ANOVA tests. For all measurements, df = 3/36. Significant differences relative to the LFD control group were demonstrated using the Dunnett's post-hoc multiple comparison test (*P < 0.05; **P < 0.01; ***P < 0.001).

## Discussion

We examined the degree to which limited early life NDEA exposure exacerbates the effects of later chronic HFD feeding on subsequent development of T2DM, NASH, and neurodegeneration. The relevance of this work to human disease is that, morbidity and mortality from major insulin-resistance diseases have escalated over the past several decades. Such trends more likely correspond with exposure rather than genetic etiologies. Our principal hypothesis is that shifts in lifestyles have led modern societies to chronically consume excess fats, and also to increase our exposures to pathogenic agents that cause insulin resistance. We focused our investigations on nitrosamines, particularly NDEA, because in previous studies, STZ, a nitrosamine-related chemical, was demonstrated to be mutagenic in high doses [17], and to cause insulin-resistance diseases at lower levels or after limited durations of exposure [18-25,76]. However, since STZ is not generally available in the environment or foodstuffs, whereas nitrosamines and related compounds are widely present in our environment and contaminate our foods, we hypothesize that limited or chronic low-level exposures to nitrosamines account for the current insulin resistance disease epidemic in the United States. Moreover, given the clear role of high dietary fat intake as a mediator of obesity, T2DM, NAFLD/NASH, and cognitive impairment, we propose that the combined effects of HFD and NDEA exposure additively promote development of insulin resistance diseases.

The knowledge that: 1) human nitrosamine exposures occur through many sources including, processed and preserved foods, tobacco smoke (direct or second hand), and nitrate-containing fertilizers; and 2) nitrosamines are mutagenic and cause tissue injury in manners similar to STZ [17,19,27,29], prompted us to consider whether nitrosamines could have pathogenic roles in our insulin resistance diseases epidemic. Although epidemiologic studies have correlated obesity and high dietary fat intake with rising rates of T2DM, NASH, and cognitive impairment [1-3,5,6,77], experimental data are somewhat varied depending on the model, and no studies have demonstrated that obesity/T2DM is sufficient to cause significant AD-type neurodegeneration with cognitive impairment. In fact, the evidence convincingly informs us that these factors alone are not sufficient, and instead serve as co-factors in the pathogenesis of neurodegeneration, including AD. Therefore, we propose that high dietary fat intake exacerbates the adverse effects of limited NDEA or other nitrosamine exposures to cause insulin resistance diseases.

We generated an experimental in vivo model in which rat pups were treated with NDEA at doses that were 5- to 500-fold lower than the cumulative doses used to cause cancer [78-81], and beginning in early adolescence, we pair-fed the rats with high (60%) or low (10%) fat content diets. The use of young rats in these studies was inspired in part by longitudinal studies of nuns demonstrating that neuro-cognitive abnormalities occur decades earlier than the onset of dementia, indicating that very early life exposures may predispose individuals to develop AD, as well as other neurodegenerative diseases [52,53], perhaps through epigenetic events such as DNA methylation or gene imprinting. Correspondingly, there is evidence that DNA methylation and other epigenetic changes in DNA increase with aging [82], and likely contribute to the pathogenesis of diseases such as diabetes and neurodegeneration [82,83]. Moreover, there is experimental evidence that nitrosamines, as well as other adduct forming toxins that contaminate foods, can mediate DNA methylation [84,85]. Since it could take years for epigenetic modifications to cause disease, and the findings in the "Nun Study" suggest that early events in life predispose individuals to develop AD [53], we utilized an experimental animal model in which low-dose NDEA exposures were administered early in life. The subsequent chronic HFD feeding during adolescence, also fits with the human disease model. Therefore, our experimental approach enabled us to examine effects of early life NDEA exposure on later cognitive function and neurodegeneration in the context of excess caloric intake, which is one of the major modifying factors correlated with insulin resistance diseases in our society.

Although the HFD feeding and NDEA treatments significantly increased body weight relative to control, and caused T2DM, characterized by fasting hyperglycemia, hyperinsulinemia, and pancreatic islet hypertrophy [86,87], the rats were not obese and they did not have hyperlipidemia. On the other hand, the NDEA ± HFD groups had steatohepatitis with hepatic insulin and/or IGF-1 resistance which were more pronounced in rats that had the combined versus individual NDEA or HFD exposures. This suggests that HFD and NDEA function additively to promote NASH and T2DM. In contrast to the C57BL/6 mouse model of HFD feeding in which NASH was associated with obesity, T2DM, and hyperlipidemia [38,39], the serological profile in the present model provided minimal evidence of hepatic insulin/IGF resistance. Since the serum ALT levels were increased in the HFD groups, perhaps biomarkers of hepatic injury in individuals with T2DM should be regarded as a potential indicator of steatohepatitis.

The NDEA treatments and HFD feedings independently caused significant deficits in spatial learning, and the combined exposures had the added effect of impairing learning and memory, again suggesting that the adverse effects were additive. Therefore, curbing either or both exposures could help prevent subsequent development of cognitive impairment. A second point is that, although the NDEA was delivered within a brief window early in life, its impact on cognitive function was sustained in the adults, similar to the effects of ic-STZ treatment [22,25]. The mechanism underlying these prolonged and possibly progressive deficits may reside in the fact that nitrosamines promote the formation of adducts that can serve as persistent sources of oxidative stress, DNA damage, and protein mal-folding or dysfunction [8,9,26,88], and ultimately lead to epigenetic changes in gene expression.

Reduced levels of ChAT expression were observed in brains of HFD, NDEA, and NDEA+HFD treated rats. In addition, NDEA exposure without HFD feeding reduced GFAP, but increased HNE and Aβ PP-Aβ, while NDEA+HFD reduced GFAP, GAPDH, Tau, pGSK-3β, AβPP-Aβ, and HNE immunoreactivities, and Tau and IL-1β mRNA, but increased pTau and the pTau/Tau ratio. Since HFD feeding alone had minimal effects on these biomarkers of neurodegeneration, the differences in reaction to NDEA versus NDEA+HFD could be attributed to interactive or amplifying effects of chronic HFD feeding/T2DM on early life, low-level NDEA exposure. This phenomenon was particularly noticeable with respect to the reductions in ChAT, which were modest in brains of rats exposed to NDEA or the HFD, but striking in brains exposed to NDEA plus HFD.

The NDEA-associated reductions in GFAP, Tau, and ChAT expression in brain are of interest because similar observations were made in humans with AD, and in the ic-STZ experimental animal model of AD-type neurodegeneration [22,25,51]. The reduced levels of GFAP suggest that glia (astrocytes) are targets of neurodegeneration. The reductions in Tau and ChAT expression are noteworthy because both genes are regulated by insulin/IGF stimulation, their expression levels are reduced in AD [51], and insulin/IGF resistance was demonstrated to be a prominent adverse effect of limited and low-level NDEA exposure resulting in neurodegeneration with cognitive dysfunction, as also occurs in AD [51]. Similarly, the relative increases in pTau, and reductions in pGSK-3β (inactive), GAPDH, and β-actin in the NDEA+HFD group reflect adverse effects of impaired insulin/IGF signaling with cytoskeletal collapse, increased oxidative stress, and reduced energy metabolism, similar to the effects of both i.c. STZ treatment in rats and sporadic AD in humans [51].

The effects of HFD, NDEA, or both exposures on AβPP and AβPP-Aβ were varied, but the most striking findings were significantly increased levels of AβPP-Aβ in NDEA-treated rats, and paradoxically decreased levels of AβPP-Aβ in brains of NDEA+HFD treated rats. Similarly, HNE immunoreactivity was also increased in brain by limited peripheral NDEA exposure, but these adverse effects of NDEA were abolished by chronic HFD feeding. The findings with respect to NDEA on AβPP-Aβ and HNE are consistent with previous observations that oxidative stress promotes AβPP-Aβ accumulation and lipid peroxidation in the CNS [89]. On the other hand, it appears that HFD feeding may have been somewhat protective, perhaps due to alterations in membrane lipid composition leading to enhanced intracellular signaling [90]. The fact that ChAT expression and cognitive function were most impaired in the NDEA+HFD group relative to control vis-à-vis low levels of AβPP-Aβ highlights the controversial role of AβPP-Aβ accumulation in relation to cognitive impairment in AD.

NDEA exposure and HFD feeding independently impaired insulin and IGF-1 signaling mechanisms in liver and brain, and in general, the combined exposures further reduced both hepatic and brain insulin and IGF-1 receptor binding compared with HFD feeding alone. Therefore, brain and hepatic insulin/IGF-1 resistance can be effectuated by either insult. Although impairments in binding to the insulin and IGF-1 receptors in brain could be explained in part by reduced expression of those receptors or receptor-bearing cells in rats treated with NDEA+HFD, generally, this was not the case for the NDEA-treated or HFD-fed groups in which the receptor expression was either elevated or similar to control. Most likely, the impaired receptor binding with attendant reduced expression of IRS signaling molecules mediated the insulin/IGF resistance. Moreover, the reductions in IGF-1 expression could account for progressive loss of IGF-1 receptor bearing cells in vivo. In some instances, ligand expression was increased, suggesting that compensatory responses had occurred due to insulin/IGF-1 resistance as occurs in T2DM.

The mechanisms of sustained brain and liver insulin- and IGF-1 resistance in the context of NDEA exposure ± HFD feeding are not well understood. The fact that NDEA ± HFD feeding caused NASH, led us to investigate whether toxic lipids stemming from NASH-related injury could contribute to NDEA-mediated neurodegeneration. Since pro-ceramide genes are increased in experimental models of NASH [68,71,74,91], and ceramides cause neurodegeneration, pro-inflammatory cytokine activation, and insulin resistance [4,38,68,71-74,92,93], we measured mRNA levels of pro-ceramide genes in liver and brain. Those studies revealed strikingly increased levels of several genes involved in ceramide generation via both biosynthesis and degradation pathways in livers of NDEA-treated rats, with virtually no additional impact of HFD feeding. Since both NDEA and ceramides are lipid soluble [94,95], and therefore likely to readily cross the blood-brain barrier, NDEA exposure could cause CNS insulin resistance and chronic injury by dual mechanisms: 1) direct neurotoxic injury with locally increased production of adducts and pro-ceramide gene expression; and 2) increased hepatic ceramide synthesis leading to the establishment of a liver-brain axis of neurodegeneration. Correspondingly, the qRT-PCR results suggest that hepatic-origin ceramide is generated by both synthetic and degradative pathways in NDEA-treated rats, whereas in the brain, ceramide gene expression was strikingly increased via degradative pathway mechanisms, and inhibited via the synthetic pathways. Preliminary studies suggest that in vivo intraperitoneal administration of toxic ceramides is sufficient to cause brain insulin resistance, neurodegeneration, and cognitive impairment (Tong, et al, unpublished).

## Conclusions

We demonstrated that limited, low-level exposure to sub-mutagenic doses of NDEA, together with chronic HFD feeding, act additively in causing peripheral, hepatic and brain insulin and IGF-1 resistance associated with T2DM, NAFLD/NASH, and neurodegeneration. The neurodegeneration had features in common with AD, including impairments in learning and memory, cholinergic function, and neuronal cytoskeletal gene and protein expression. These abnormalities were associated with increased pro-ceramide gene expression, and since ceramides promote insulin resistance, neuro-inflammation, and neurodegeneration, characterization and measurement of ceramides and related lipotoxins in blood may prove useful as biomarkers of insulin-resistance disease progression toward T2DM, NASH, or AD. The molecular and biochemical abnormalities detected are quite reminiscent of the findings in the ic-STZ model [51]. Altogether, these results raise concerns about the double insult of chronic, low-level nitrosamine exposure and high dietary fat consumption as major mediators of our insulin resistance disease epidemic.

## Abbreviations

AβPP: amyloid-β-precursor protein; AβPP-Aβ: amyloid-β peptide; AChE: acetylcholinesterase; AD: Alzheimer's disease; CER: Ceramide synthase; ChAT: choline acetyltransferase; ELISA: enzyme-linked immunosorbant assay; GFAP: glial fibrillary acidic protein; GSK-3β-glycogen synthase kinase-3β; H&E: hematoxylin and eosin; HFD: high fat diet; HNE: 4-hydroxy-2-nonenal; HRP: horseradish peroxidase; i.p.: intraperitoneal; IGF: Insulin like growth factor; IRS: Insulin receptor substrate; LFD: low fat diet; LHE: Luxol fast blue hematoxylin and eosin; MCI: mild cognitive impairment; NASH: non-alcoholic steatohepatitis; NDEA: N-nitrosodiethylamine; NDMA: N-nitrosodimethylamine; P3: postnatal day 3; qRT-PCR: quantitative reverse transcriptase polymerase chain reaction; SMPD: sphingomyelin phosphodiesterase; SPTLC: Serine palmitoyltransferase; STZ: Streptozotocin; T2DM: Type 2 diabetes mellitus; TBS: Tris buffered saline; UGCG: UDP-glucose ceramide glycoysltransferase.

## Competing interests

The authors declare that they have no competing interests.

## Authors' contributions

All authors have read and approved of the final manuscript. Ming Tong generated the model, harvested and processed tissue for histopathologic, molecular, and biochemical assays, performed qRT-PCR, ELISA, and immunostaining studies, analyzed data, reviewed manuscript. Lisa Longato and Margot Lawton performed serum assays, ELISAs, and qRT-PCR assays, analyzed data, reviewed manuscript Suzanne de la Monte-generated hypothesis, designed experiments, supervised work and data analysis, interpreted results, wrote manuscript.
